# Phase II Study of Pomalidomide in Patients with Castration-Resistant Prostate Cancer

**DOI:** 10.3390/cancers3033449

**Published:** 2011-09-02

**Authors:** Robert J. Amato, L. Michael Glode, Jeremy Podolnick, Robert Knight, David Crawford

**Affiliations:** 1 Memorial Hermann Cancer Center, University of Texas, 6410 Fannin Street, Suite 830, Houston, TX 77030, USA; 2 Health Science Center, University of Colorado, Denver, CO 80217, USA; E-Mails: mike.glode@ucdenver.edu (M.G.); david.crawford@uchsc.edu (D.C.); 3 University of Colorado Cancer Center, Aurora, CO 80045, USA; 4 Celgene Corporation, Summit, NJ 07901-3915, USA; E-Mail: rknight@celgene.com

**Keywords:** prostate cancer, metastatic, castration-resistant, pomalidomide, thalidomide analog, antiangiogenic, immunomodulatory

## Abstract

Pomalidomide is a distinct immunomodulatory agent that also displays anti-proliferative and proapoptotic activity. The purpose of this study was to assess the efficacy and safety of pomalidomide for the treatment of chemotherapy-naïve patients with metastatic castration-resistant prostate cancer (CRPC). *Methods*: Pomalidomide was administered orally in doses of 1 or 2 mg/day without interruption. Follow ups were conducted every 4 weeks with evaluation of study outcomes at 12 weeks. The principal study outcomes were PSA response, time to progression (TTP) using RECIST, overall survival (OS), and safety. A total of 32 patients were enrolled: 15 in the 1 mg/day cohort (median baseline PSA level of 12.30 ng/mL [0.8–236.0]), and 17 in the 2 mg/day cohort (median baseline PSA level of 12.50 ng/mL [0.6–191.8]). *Results*: In the 1 mg cohort disease was stabilized for ≥28 days in eight patients, and median TTP was 2.90 months. In the 2 mg cohort, PSA decreased ≥50% in three patients, disease was stabilized for ≥28 days in seven patients, and median TTP was 5.87 months. Toxicity in both cohorts was predominantly grade 1 or 2; 2 grade 3 toxicity (fatigue) occurred in the 1 mg cohort, and 5 grade 3 toxicities (chest pain, diarrhea, epigastric pain, impaction, pain) occurred in the 2 mg cohort. One grade 4 toxicity of cardiac ischemia occurred. *Conclusions*: Pomalidomide shows promising activity in patients with CRPC and has an acceptable safety profile.

## 1. Introduction

Prostate cancer is the most common malignancy and the second leading cause of cancer death among men in the United States; in 2010 an estimated 217,730 new cases were diagnosed and 32,050 men were expected to die from the disease [1]. Although most cases are diagnosed at a local stage, approximately 4% of patients present with metastatic disease [2]. Other patients ultimately develop metastasis despite previous surgery, radiation therapy, and androgen deprivation. The progression to castration-resistant prostate cancer (CRPC) marks a clinical acceleration of disease associated with a poor outcome. First-line treatment for CRPC consists of docetaxel in combination with prednisone or estramustine [3]. Recently, the drug abiraterone received Food and Drug Administration approval for treating docetaxel-refractory disease [4]. Despite high response rates to these regimens, patients will eventually develop progressive disease, with a median time to progression of 6–8 months and a median survival time of only 17–19 months [5-7]. Although cabazitaxel has recently been approved as a second-line treatment for CRPC [8], no consensus exists regarding the best approach following docetaxel failure [9]. Current options include second line-hormonal, taxane, immunotherapy or novel agents in research trials. There continues to be a need for additional therapies in this setting, with targeted therapy representing a potential viable option.

Thalidomide is a potentially effective therapy for patients with CRPC. In addition to having antiangiogenic properties [10,11], it has been shown to affect adhesion molecule expression; T-cell and NK cell co-stimulation; stimulation of IL4, IL5, IL-10 and IL-12; and down-regulation of TNF-α, IL-6, and COX-2 synthesis [7,8]. Findings from phase II clinical trials of thalidomide monotherapy and combination therapy with docetaxel indicate that objective tumor responses and PSA responses have been achieved in CRPC patients [12-14].

Lenalidomide is a thalidomide derivative that has been shown by *in vitro* studies to be 50 to 2,000 times more potent than thalidomide in inhibiting TNF-α production and stimulating the proliferation of T-cells, and it is 50 to 100 times more potent than thalidomide in augmenting the production of IL-2 and IFN-γ. Lenalidomide is strongly antiangiogenic, although the exact mechanism for the antiangiogenic effect is still unclear. Because it is significantly less neurotoxic than its parent thalidomide, it was evaluated in prostate cancer. Thirty-five patients with castration-resistant prostate carcinoma were treated, no single agent activity had been observed [15,16]. A phase I combination of lenalidomide and docetaxel for patients with metastatic CRPC evaluating decline in PSA found the combination to be safe with evidence of treatment response, which prompted cohort expansion to 10 patients. [17] Thus, a Phase III study is currently comparing docetaxel and prednisone with or without lenalidomide. A study evaluating the safety of a 6 month lenalidomide treatment in non-metastatic prostate cancer found that it has an acceptable toxicity profile with long-term disease stabilization and PSA declines [18].

Pomalidomide (Figure 1) is a distinct immunomodulatory agent that also displays anti-proliferative and pro-apoptotic activity in multiple myeloma [19-26]. In a phase I trial in 24 patients with relapsed or refractory multiple myeloma, pomalidomide treatment resulted in a >25% reduction in M-protein level in 67% of patients, with 13 patients (54%) achieving a >50% reduction, and four patients (17%) achieving complete remission [27]. The clinical response was accompanied by significantly increased serum IL-2 receptor and IL-12 levels, consistent with activation of T-cells, monocytes, and macrophages.

The current study investigated the efficacy and safety of single-agent pomalidomide for the treatment of CRPC, as indicated by PSA response, time to progression (TTP), and adverse events evaluated according to the National Cancer Institute Common Toxicity Criteria for Adverse Events (NCI CTCAE) version 3.0 [28].

## 2. Methods

### 2.1. Study Design

This was a phase II open-label study of the efficacy and safety of pomalidomide administered orally in doses of 1 mg/day or 2 mg/day to men with CRPC prior to docetaxel. The first 15 patients recruited into the study received 1 mg/day and were maintained on this dosage as long as they had no unacceptable toxicity and did not meet the criteria for progressive disease. Because of the low toxicity profile of the drug in this patient cohort, the study was amended to allow the remaining 17 patients to receive 2 mg/day. Patients were followed every 4 weeks, and treatment results were evaluated at 12 weeks. The study protocol was approved by both Institutional Review Boards.

### 2.2. Patients

The study population consisted of patients who presented to one of the two study sites for treatment of CRPC. Nineteen patients were assigned to the initial 1 mg cohort. If there were three or fewer responses, no further study would be considered. If at least four responses were elicited, a maximum of 17 additional patients would be added to the second stage cohort. Per the protocol, a minimum of 36 patients would be treated. Inclusion requirements for the study were: histological confirmation of adenocarcinoma of the prostate with radiographic evidence of progressive metastases and/or PSA progression (two consecutive rises of at least 1 ng/mL separated by at least 28 days); maintain luteinizing hormone releasing hormone or gonadotropin releasing hormone antagonist therapy with testosterone level of ≤50 ng/mL; must have stopped anti-androgen therapy for at least 6 weeks (*i.e.*, bicalutamide) prior to entering the study; and adequate physiology as assessed by standard laboratory tests for hepatocellular, renal function, and hematologic. Patients were excluded from the study for: any condition that would prevent the patient from signing the informed consent form, any condition that would place the patient at unacceptable risk or confound the ability to interpret study data, use of any other experimental drug or therapy within 28 days, tumors containing small cell or sarcomatoid elements, symptomatic bone metastases, prior use of any thalidomide-type drug, concurrent use of other anticancer agents, or non-PSA producing tumors. All patients provided written informed consent to participate in the study.

### 2.3. Intervention

Pomalidomide was supplied to all patients as 1 mg capsules by the study sponsor (Celegene Corp., Warren, NJ, USA). The drug was dispensed every 28 days, and patients were instructed to take it in the morning at approximately the same time each day. Filgrastim (G-CSF) was permitted for the treatment of neutropenia. Concurrent chemotherapy or immunotherapies were prohibited during the study. Treatment with pomalidomide was discontinued for patient choice, serious adverse events, protocol violation, progression of disease, death, or patient lost to follow-up.

### 2.4. Study Endpoints

The primary study endpoint was objective PSA response (≤50% decline). The secondary study endpoints were TTP including PSA progression and RECIST for radiologic progression, OS, and safety.

### 2.5. Study Assessments

#### 2.5.1. Pretreatment Evaluations

The baseline evaluation included a patient history, physical examination, chest x-ray, computed tomography of the abdomen and pelvis, bone scan, assessment of Zubrod performance status, cardiac profile including electrocardiography, hematologic profile (*i.e.*, complete blood count with differential and platelets), coagulation profile (*i.e.*, prothrombin time and partial thromboplastin time), biochemical profile (*i.e.*, total protein, uric acid, blood urea nitrogen, creatinine, lactate dehydrogenase, aspartate aminotransferase, alanine aminotransferase, alkaline phosphatase, phosphorus, total bilirubin, sodium, potassium, CO2, chloride, calcium, albumin, and glucose), thyroid function studies, testosterone, and urinalysis.

#### 2.5.2. Efficacy Assessment

The major objective was to assess PSA response to treatment with pomalidomide and to determine if the drug is worthy of further study. PSA levels were assessed every 4 weeks. A decline in PSA level of ≥50% from baseline lasting at least 28 days was considered to be of clinical interest. This surrogate marker was shown by Small *et al.* to be significantly associated with a prolonged median OS and TTP [31]. TTP was defined as the time between the drug start date and the time that disease progression or patient death. Overall survival was defined as the time between drug start date and till death or last follow-up. Patients lost to follow up were censored and not included as events.

#### 2.5.3. Safety Assessments

Safety was assessed every 4 weeks in all patients who received at least 1 dose of the study drug. All toxic adverse events and laboratory abnormalities were noted and graded for severity whenever possible according to NCI CTCAE version 3.0. Patients experiencing treatment-related ≥grade 3 toxicity had their study drug interrupted until toxicity resolved to ≤grade 2 and then would restart at the same dose.

#### 2.5.4. Statistical Analysis

Laboratory data were analyzed and tabulated in terms of frequency of the worst severity grade observed (NCI CTCAE). TTP was defined as the number of days from the day the patient started study drug to the date of the patient's tumor progression or death. All events of tumor progression were included, regardless of whether the event occurred while the patient was still taking the study drug or after the patient discontinued the study drug. Overall survival was calculated from the day the patient started study drug to the date of the patient's death or last follow-up. Both of the analyses were estimated using Kaplan-Meier methodology. GraphPad Prism 5.04 was used for the analysis.

## 3. Results

### 3.1. Recruitment and Follow-Up

Eligible patients were recruited between December 2003 and July 2004. All patients included in the study were evaluated at baseline (≤14 days before the first study dose) and once every 4 weeks for PSA response and safety. Patients remained on therapy continuously until documented disease progression.

### 3.2. Patient Characteristics

A total of 32 patients were recruited into the study and treated with pomalidomide, 15 patients in the 1 mg/day dose cohort and 17 patients in the 2 mg/day dose cohort. (Table 1) One patient in the 2 mg cohort was lost to follow-up before 4 weeks and was not included in the analysis. The median ages in the two treatment cohorts were similar: 75 (range 46–92) in the 1 mg cohort and 74 (range 56–85) in the 2 mg cohort. The median baseline PSA level was similar in the 1 mg cohort as well as the 2 mg cohort (12.3 and 12.5, respectively). Of the 32 patients, 34% were treated with radiation only, 17% with prostatectomy only, and 34% with both radiation and prostatectomy. No patients received docetaxel as prior treatment. The majority of metastases in the 1 mg cohort were bone only in seven patients (46%). Of the remaining patients, four patients (27%) having bone and nodal metastases, three (20%) patients had only nodal metastases, and one patient (7%) had no visible metastasis, but rising PSA. In contrast, metastases in patients from the 2 mg cohort were about equally distributed between bone only in nine patients (53%) and bone and nodal in two patients (12%), with the remainder observed as no visible metastasis, but rising PSA (24%) and nodal (12%). In the 1 mg cohort, 10 patients completed 12 weeks of therapy, and one patient remained on the study for a total of 68 weeks. In the 2 mg cohort, 16 patients completed 12 weeks of therapy, and one patient remained on study for total of 59 weeks.

### 3.3. Efficacy

The efficacy of the two drug doses in reducing PSA levels appeared to be comparable with 53% of patients in the 1 mg cohort and 41% of patients in the 2 mg cohort showing either PSA reduction or stable disease (Table 2).

Overall, two of the 32 evaluable patients (6%) had a ≥50% reduction in PSA values, and an additional nine patients (28%) showed a <50% reduction in PSA values. Stable disease, which includes PSA reduction <50%, was seen in 14 out of 32 patients (44%). Overall survival was 34.17 months for the 1 mg cohort and 46.97 months for the 2 mg cohort (Figure 1 and Table 3). The median TTP was 5.9 months in the 2 mg cohort and 2.9 months in the 1 mg cohort.

### 3.4. Safety

The most common treatment-related adverse event in the 1 mg cohort was fatigue, which affected eight out of 15 patients (53%; 5 grade 1, 1 grade 2, and 2 grade 3). There were no other grade 3 toxicities in this cohort. Other common grade 1 or grade 2 toxicities seen at the 1 mg/day dose were myalgia (40%), constipation (33%), and paresthesia (33%). In the 2 mg/day cohort, the most common treatment-related adverse events were rash, which affected nine out of 17 patients (53%; NCI CTCAE grade 1 and 2) and pain, which affected nine out of 17 patients (53%; NCI CTCAE grade 1, 2, and 3). One grade 4 toxicity appeared in this cohort with cardiac ischemia and five other grade 3 toxicities included chest pain, diarrhea, epigastric pain, impaction, and pain (Table 4).

### 3.5. Discussion

This phase 2 study evaluated single-agent therapy using pomalidomide in patients with progressive CRPC prior to docetaxel chemotherapy. The results of this trial demonstrate that pomalidomide may stabilize disease progression in patients with CRPC. There is no way to compare the efficacy at 1 mg dose *vs.* 2 mg dose; however, the activity found at these two levels is of sufficient interest to warrant further study of both the subject drug and the class in prostate cancer. Pomalidomide was generally well tolerated. The most common side effects were typically mild or moderate in severity independent of the dose cohort and included fatigue, paresthesia, and constipation. The safety profile observed in this patient population is consistent with that reported in a phase I trial of patients with refractory multiple myeloma.

A single objective response was observed. Forty-six percent of patients treated with pomalidomide had no disease progression based on RECIST guidelines, and 6% had a greater than 50% PSA response. In the 1 mg cohort, 6% of patients had rising PSA only with no visible metastasis, and in the 2 mg cohort, this number was 24%. However, this may not indicate that high dose is more effective, but rather it could reflect the higher percentage of PSA recurrence only patients who did not have visible metastasis in the 2 mg cohort compared to the 1 mg cohort. In addition, 44% of all patients maintained stable PSA levels for a median of 4.4 months. The changes in PSA values may depend on multiple factors that do not necessarily correlate with radiologic assessments. This may be particularly true for cytostatic agents, which may modulate PSA levels independently of influencing tumor cell growth or survival. The primary endpoint was not met as PSA declined in only 6% of patients. However, pomalidomide, like its parent compound thalidomide, differs from cytotoxic chemotherapeutic drugs in that they typically produce cytostatic effects. Consequently, pomalidomide was expected to stabilize the disease rather than demonstrate tumor shrinkage. Therefore, clinical benefit, defined as a delay in TTP, may be a more appropriate measure of the antitumor activity of pomalidomide rather than classical response assessment based on RECIST that was established to evaluate cytotoxic agents.

Pomalidomide in combination with taxane is a potential option to be considered to further evaluate pomalidomide in CRPC patients. In addition, studies of pomalidomide as second-line therapy in combination with an anthracycline, or a platinum analogue could be considered. Furthermore, the parent drug thalidomide has been developed as a monotherapy treatment option for other solid tumors and hematological malignancies, and pomalidomide has the potential to be developed in a similar manner.

The potential of intervaccination combination strategies is of considerable interest. With evidence of tumor response via RECIST and PSA reduction, combining pomalidomide with other strategies that demonstrate increase in overall survival without evidence of tumor response may be of particular interest. For example, combining pomalidomide with the sipuleucel-T, a vaccine strategy recently approved for the treatment of prostate cancer, may be successful in treating CRPC. CRPC patients treated with sipuleucel-T have shown to have delayed disease progression and increase in overall survival despite no evidence of tumor response indicated by RECIST or lowered levels of PSA [29]. This this may have resulted from a possible class effect or some other previously unknown feature of prostate cancer [29,30]. Bevacizumab may also be of interest considering one study that examined bevacizumab in combination with other strategies showed an increase in progression-free survival and reduction of PSA levels in comparison to bevacizumab alone [31]. Other anti-vascular endothelial growth factor approaches may be effective as well, and further research should be conducted using combination strategies.

## 4. Conclusions

Results from this trial suggest that pomalidomide 1 mg or 2 mg administered daily stabilized disease in nearly half of the patients with CRPC. Pomalidomide was generally well tolerated in this patient population and had a safety profile similar to that observed in patients with advanced CRPC. Results in this study measured PSA only disease *versus* visible metastasis. Future clinical trials in patients with CRPC could assess the benefit and safety profile pomalidomide in combination with chemotherapy.

## Figures and Tables

**Figure 1.**
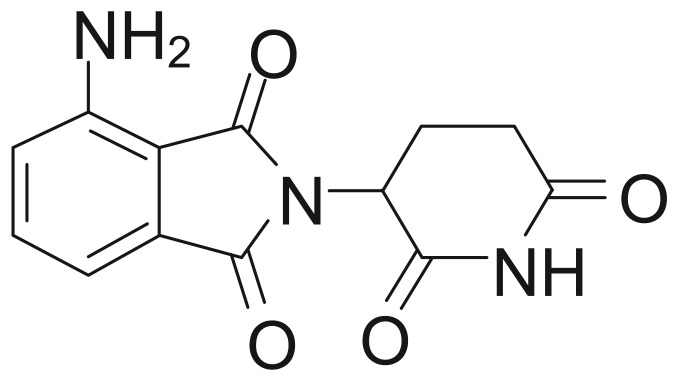
Chemical compound of pamolidomide.

**Figure 1.**
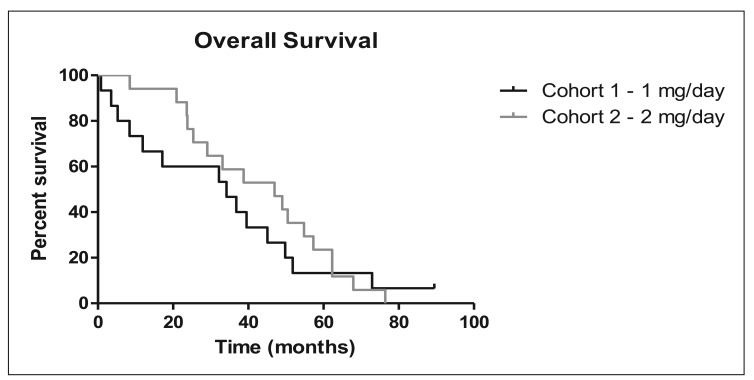
Overall Survival.

**Table 1.** Patient characteristics.

**Patient Characteristics**	**1 mg No. (%)**	**2 mg No. (%)**
Age range	46-92	56-85
Median age	75	74
White	15 (100)	15 (88)
African American	0	2 (11)
**Prior Local Therapy**		
Radiation only	7 (46)	4 (23)
Prostatectomy only	3 (20)	2 (11)
Radiation + Prostatectomy	1 (6)	8 (47)
None	4 (26)	3 (17)
**Gleason**		
<6	2 (13)	1 (6)
7	5 (33)	7 (41)
8	5 (33)	2 (12)
9	2 (13)	6 (35)
10	1 (7)	0
Not available	0	1 (6)
**Metastatic sites**		
Bone disease <3	2 (13)	6 (35)
Bone disease >3	5 (33)	3 (17)
Soft tissue disease	3 (20)	2 (12)
Bone & soft tissue	4 (26)	2 (12)
Rising PSA Only	1 (6)	4 (24)

**Table 2.** Results of pomalidomide treatment.

**Best PSA Response**	**1 mg No. (%)**	**2 mg No. (%)**
<15 %	1 (7)	1 (6)
>15 %	2 (13)	2 (12)
>30 %	0	2 (12)
>45 %	1 (7)	0
>50 %	0	0
>60 %	0	2 (12)
>80 %	0	0
>90 %	0	1 (6)

Rise	11 (73)	9 (52)
**Best Tumor Response**		
Complete Response	0	0
Partial Response	1 (6)	0
Stable Disease	7 (46)	7 (41)
Progressive Disease	6 (42)	4 (24)
No Mets	0	5 (29)
Not Evaluable	1 (6)	1 (6)
**PSA**		
Baseline PSA	0.80–236.0	0.60–191.8
Median Baseline PSA	12.3	12.8
**Survival (months)**		
PSA TTP Range	0.83–16.80	2.77–13.73
PSA TTP Median	2.90	5.90
Radiographic TTP Range	0.83–39.50	2.77–37.80
Radiographic TTP Median	3.07	5.90
OS Range	0.83–89.47+	8.47–76.40
OS Median	34.17+	46.97

PSA, prostate-specific antigen; TTP, time to progression; OS, overall survival.

**Table 3.** Overall Survival.

	**Cohort 1 – 1 mg/day**	**Cohort 2 – 2 mg/day**
Total Patients	15	17
Total Surviving (Censored)	1	0
Total Deceased	14	17
Median	34.17+ (0.83 – 89.47+) months	46.97 (8.47 – 76.4) months

**Table 4.** Grade 3 and 4 adverse events for pomalidomide.

	**Grade 3**	**Grade 4**
**1 mg cohort (N = 15)**		
Fatigue	2	
**2 mg cohort (N = 17)**		
Cardiac ischemia		1
Chest pain	1	
Diarrhea	1	
Epigastric pain	1	
Impaction	1	
Pain	1	
